# Adjuvant and Salvage Radiotherapy after Prostatectomy: A Systematic Review and Meta-Analysis

**DOI:** 10.1371/journal.pone.0104918

**Published:** 2014-08-14

**Authors:** Changhao Chen, Tianxin Lin, Yu Zhou, Doudou Li, Kewei Xu, Zhihua Li, Xinxiang Fan, Guangzheng Zhong, Wang He, Xu Chen, Xianyin He, Jian Huang

**Affiliations:** 1 Department of Urology, Sun Yat-sen Memorial Hospital, Guangzhou, China; 2 Department of Hepatobiliary Surgery, Sun Yat-sen Memorial Hospital, Guangzhou, China; 3 Department of Oncology, Sun Yat-sen Memorial Hospital, Guangzhou, China; 4 Department of Medical Statistics and Epidemiology, School of Public Health, Sun Yat-sen University, Guangzhou, China; University of Kentucky College of Medicine, United States of America

## Abstract

**Purpose:**

In men with adverse prognostic factors (APFs) after radical prostatectomy (RP), the most appropriate timing to administer radiotherapy remains a subject for debate. We conducted a systemic review and meta-analysis to evaluate the therapeutic strategies: adjuvant radiotherapy (ART) and salvage radiotherapy (SRT).

**Materials and Methods:**

We comprehensively searched PubMed, EMBASE, Web of Science and the Cochrane Library and performed the meta-analysis of all randomized controlled trials (RCTs) and retrospective comparative studies assessing the prognostic factors of ART and SRT.

**Results:**

Between May 1998 and July 2012, 2 matched control studies and 16 retrospective studies including a total of 2629 cases were identified (1404 cases for ART and 1185 cases for SRT). 5-year biochemical failure free survival (BFFS) for ART was longer than that for SRT (Hazard Ratio [HR]: 0.37; 95% CI, 0.30–0.46; p<0.00001, I^2^ = 0%). 3-year BFFS was significantly longer in the ART (HR: 0.38; 95% CI, 0.28–0.52; p<0.00001, I^2^ = 0%). Overall survival (OS) was also better in the ART (RR: 0.53; 95% CI, 0.41–0.68; p<0.00001, I^2^ = 0%), as did disease free survival (DFS) (RR: 0.53; 95% CI, 0.43–0.66; p<0.00001, I^2^ = 0%). Exploratory subgroup analysis and sensitivity analysis revealed the similar results with original analysis.

**Conclusion:**

ART therapy offers a safe and efficient alternative to SRT with longer 3-year and 5-year BFFS, better OS and DFS. Our recommendation is to suggest ART for patients with APFs and may reduce the need for SRT. Given the inherent limitations of the included studies, future well-designed RCTs are awaited to confirm and update this analysis.

## Introduction

Radical prostatectomy (RP) is a standard and highly effective care treatment for selected patients with prostate cancer assuming with favorable prognostic features [1]. After radical prostatectomy the men with adverse pathological factors (APFs) such as positive surgical margins, seminal vesicle invasion, extra prostatic extension and higher Gleason scores are advised administering radiotherapy [2], [3]. In terms of efficacy, prognostic factors and toxicity, the two therapeutic strategies are used: immediate postoperative radiotherapy or adjuvant radiotherapy (ART) and delay postoperative radiotherapy or salvage radiotherapy (SRT) [1], [4]. ART is the administration of radiotherapy post-prostatectomy to patients at a higher risk of recurrence due to APFs prior to evidence of disease recurrence, while SRT is the administration of radiotherapy to the prostatic bed and possibly to the surrounding tissues, including lymph nodes, in the patients with prostate specific antigen (PSA) recurrence after surgery but no evidence of distant metastatic disease [5], [6]. Delivery of the ART or SRT becomes both therapeutic and diagnostic; PSA response indicates local persistence or recurrence [7], [8]. However, when a biochemical recurrence occurred, in the absence of a detectable recurrence, it is hard to distinguish the local recurrence in prostatic bed from distant metastases [9]. A direct comparison evidence of adjuvant and salvage RT is difficult to find due to the numerous confusing factors [2]. Although several studies comparing ART and SRT have been reported, most are small series with unclear results [10], [11], [12], [13], [14], [15]. The appropriate timing of postoperative RT, either early in the adjuvant setting, or after PSA recurrence in the salvage setting, remains unclear [16], [17]. Therefore, we systemically searched and analyzed the available literatures to evaluate the efficiency, safety, and potential advantages of ART and SRT.

## Materials and Methods

### 1. Search strategy

A literature search was performed in February 2013. The primary sources were the electronic databases of PubMed, EMBASE, MEDLINE, Web of Science and the Cochrane Library. The following MeSH terms and their combinations were searched in [Title/Abstract]: [(ART/adjuvant radiotherapy/immediate postoperative radiotherapy/adjuvant RT) and (SRT/salvage radiotherapy/Postoperative radiotherapy/salvage RT) and (prostate cancer/prostatectomy/radical prostatectomy)]. In addition, the reference lists of relevant articles were manually searched to find other potentially eligible studies. References of systematic reviews identified in the background search and references of eligible studies were hand searched. No language restriction was imposed and only the most recent publication was included when duplicates were identified.

### 2. Selection criteria

If either reviewer felt a title and abstract met study eligibility criteria, the full text of the study was retrieved. References of systematic reviews identified in the background search and references of eligible studies were hand searched. The full manuscripts of all articles identified in the search were screened for eligibility criteria by 2 reviewers (Yu Zhou and Tianxin Lin) using a standardized form. Disagreements were resolved through discussion.

The eligibility criteria in the ART arm were as follows: 1. Patients must have at least one of the following risk factors: 1) Positive margins; 2) Extra prostatic extension with or without seminal vesicle invasion; 3) lymph node invasion. 2. Patients were irradiated within 6 months of the RP; 3. Patients had an undetectable serum PSA at the start of RT.4. None received any neoadjuvant therapy. The eligibility criteria of SRT arm defined as: 1. Patients were referred for RT because of a persistent postoperative serum PSA.2. Patients manifested a PSA recurrence after a period of undetectable PSA. Articles were excluded based on the following criteria: (1) letters or review articles, (2) laboratory studies, (3) case reports and animal experimental studies, (4) absence of key information such as sample size, hazard ratio (HR) and risk ratio (RR), 95% CI, and P value, 5) the outcomes of interest (as BFFS, OS etc.) were impossible to calculate or the standard deviation and confidence interval of the tested parameters were not reported (Table S1 in File S1).

### 3. Quality assessments

Data from the included studies were systematically assessed the quality of all the studies included by two of the authors (Changhao Chen and Doudou Li) that double-checked by both. Any disagreement was resolved by the adjudicating senior authors (Jian Huang). We evaluated the studies for the level of evidence provided according to criteria by the Centre for Evidence-Based Medicine in Oxford, UK [18]. The methodological quality of retrospective studies was rated by the modified Newcastle-Ottawa scale [19], [20]. The scale focuses on three factors: patient selection, comparability of the study groups, and assessment of outcome. We allot the score of 0–9 (allocated as stars) for each study. RCTs and retrospective studies achieving six or more stars were considered to be of high quality (Table S2 in File S1).

### 4. Data extraction and outcomes of interest

Two investigators (Wang He and Yu Zhou) searched the publications independently using standardized data-abstraction forms. When the two investigators discovered different results, an independent expert (Tianxin Lin) in oncology made the final decision of study conclusions. Information collected from these publications included first author, year of publication, targeted treatment, number of patients, patient characteristics, study design (blinded or not), and the outcomes.

The primary outcomes were 5-year BFFS, 3-year BFFS, OS, and DFS. Biochemical recurrence as a detectable or rising PSA value after surgery that is >0.2 ng/ml with a second confirmatory level >0.2 ng/ml. OS of included studies is defined as the time from random assignment to death, irrespective of the cause of death. DFS is defined as the duration of time from random assignment to documented disease relapse or death, whichever occurs first. The secondary outcomes were Metastasis-free survival (MFS).

### 5. Statistical analysis

The Meta-analyses were carried out using Review Manager Version 5.2 software (Review Manager, Version 5.2 for Windows, The Cochrane Collaboration, 2013). The hazard ratio (HR) was used as a summary statistic for long-term outcomes (survival analysis) as described by Parmar et al [21]. An HR of less than 1 represented a survival benefit favoring the simultaneous group. Medians were converted to means using the technique described by Hozo et al [22]. The reported risk ratio (RR) and mean difference (MD) with 95% confidence interval (CI) were used in the analysis. Heterogeneity was assessed with I^2^ statistics. An I^2^ value of more than 75% was considered to indicate high statistical heterogeneity. Reasons for statistical heterogeneity were explored using sensitivity analyses (exclusion of individual studies). The random-effects model was used if there was heterogeneity between studies; otherwise, the fixed-effects model was used [23]. Simple linear regression was utilized to identify factors associated with 5-year BFFS rate between ART and SRT. Variables tested included median radiation dose, median preoperative PSA and median pre-RT PSA [24]. Prespecified subgroup analyses were performed according to district, patient age, radiation dose and publication year to evaluate ART and SRT. All subgroup analyses followed the same meta-analysis procedure. Sensitivity analyses were performed for high quality studies. Funnel plots were used to screen for potential publication bias.

## Results

### 1. Data retrieval

**Figure 1:**
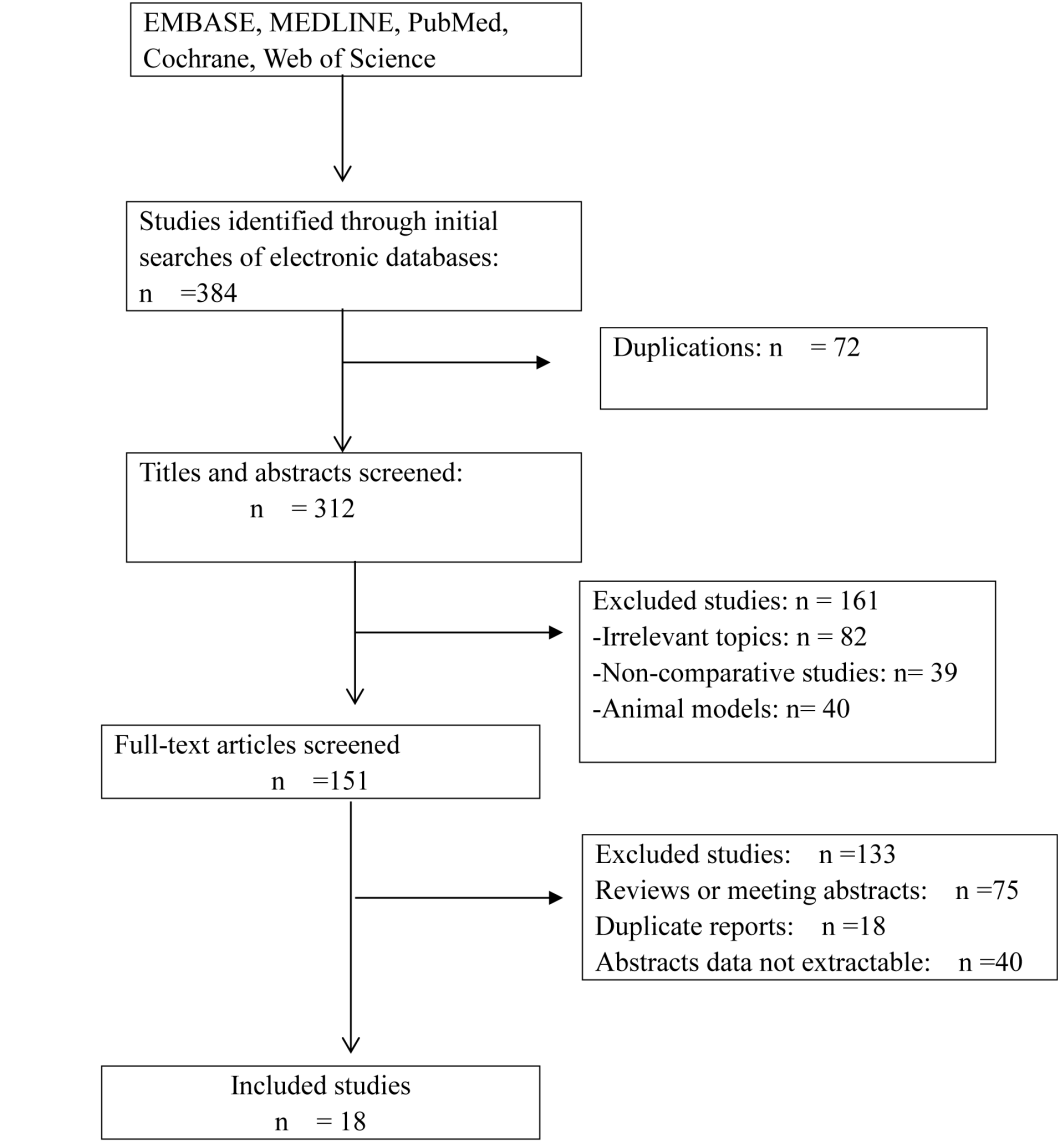
Flow diagram of studies identified, included and excluded.

Eighteen studies including 2629 cases (1416 cases for ART and 1213 cases for SRT) fulfilled the predefined inclusion criteria and were included in the final analysis (Figure 1). All studies were full-text articles [2], [25], [26], [27], [28], [29], [30], [31], [32], [33], [34], [35], [36], [37], [38], [39], [40], [41], [42]. Examination of the references listed for these studies and for the review articles did not yield any further studies for evaluation. Agreement between the three reviewers was 95% for study selection and the quality assessment of trials.

### 2. Study characteristics

The characteristics of included studies are indicated in Table 1. Among the included studies, there was no RCT between ART and SRT. Two retrospective studies were matched in a 1∶1 ratio according to preoperative prostate-specific antigen Gleason score, seminal vesicle invasion, surgical margin status, and follow-up from date of surgery [38], [39] (level of evidence: 3a); and 9 retrospective studies were retrospective studies compared high-risk series of patients (level of evidence: 3b) [2], [27], [30], [31], [32], [34], [35], [36], [41], [42]; 7 retrospective studies used no distinction between risk series of patients (level of evidence: 4) [25], [26], [28], [29], [33], [37], [40].

**Table 1:** Study characteristics.

Reference	Year	SampleSize	InclusionPeriod	Country/District	Follow-up(month)	Studytype	Level ofevidence	Qualityscore
Hudsonet al.[29]	2008	40	2002–2007	England	34(25–47)	RET	3b	★★★★★★
Wadasakiet al.[30]	2006	57	1997–2004	Japan	33(12–98)	RET	3b	★★★★★★
TAYLORet al.[2]	2003	146	1987–1998	United States	53 (ART: 68; SRT: 35)	RET	3b	★★★★★★
HAGANet al.[34]	2004	157	1989–1997	United States	ART: 53.3(12–104);SRT: 66.4(7–331)	RET	3b	★★★★★★
Dettiet al.[39]	2012	307	1995–2010	Italy	ART: 39.6(15.6–159.6)SRT: 54(19.2–135.6)	RET	3b	★★★★★★
OSTet al.[36]	2010	178	1999–2009	Belgium	36(3–120)	Matched control	3a	★★★★★★
Budihartoet al.[38]	2010	219	1991–2004	Belgium.	ART: 103.5(55–190)SRT: 121(60–194)	RET	4	★★★★★
Trabulsiet al.[37]	2008	192	1987–2002	United States	ART: 94(26–190)SRT: 97(30–207)	Matched control	3a	★★★★★
BARBARA.et al.[40]	2008	431	1996–2006	Italy	ART: 32(0–129)SRT: 97(0–108)	RET	3b	★★★★★★
PACHOLKEet al.[35]	2004	163	1982–2000	United States	ART: 70(4–153)SRT: 69(2–167)	RET	4	★★★★★
Sasakiet al.[33]	2006	105	1996–2004	Japan	23(3–68)	RET	4	★★★★
Tsienet al.[32]	2003	95	1986–1997	United States	ART: 121.2(57.6–174)SRT: 105.6(24–204)	RET	3b	★★★★★★
Doet al.[25]	2002	115	1987–1996	United States	ART: 90.8(26–157)SRT: 86.6(20–149)	RET	3b	★★★★★★
Cattonet al.[24]	2001	11	1987–1994	Canada	44.4(2.4–108)	RET	4	★★★★★
Valicentiet al.[23]	1998	79	1992–1997	United States	39	RET	4	★★★★★
Viciniet al.[27]	1999	61	1987–1993	United States	ART: 49(21–100)SRT: 46(8–78)	RET	4	★★★★
Nudellet al.[28]	1999	105	1993–1998	United States	ART: 38.3 SRT: 67.3	RET	3b	★★★★★
Mayeret al.[26]	2002	66	1987–1999	Austria	ART: 58.5(6.7–141.5)SRT: 56.4(8.4–115.2)	RET	4	★★★★

ART = adjuvant radiotherapy; SRT = salvage radiotherapy; RET = retrospective.

Included trials were published between 1982 and 2010. The selected trials were conducted in United States (9), Italy (2), Belgium (2), Japan (2), Canada (1), Australia (1), and England (1). A total of 2629 randomized participants were included and the sample size ranged from 40 to 431 patients. The follow-up time ranged from the day of discharge to 204 months in Table 2.

**Table 2:** Characteristics of included studies.

Reference	Group	N	Age	Gleasonscore≤6/7/≥8	PostoperativestagepT2/pT3/pT4	Radiationdose (Gy)	3D-CRT	Interval(months)	preoperativePSA(ng/ml)	Pre-RT PSA(ng/ml)	5-yearBFFS (%)	SVIN (%)	ECEN(%)	PSMN(%)	PreoperativehormonesN(%)	PostoperativeHormonesN(%)
Hudsonet al.[29]	ART	12	64(59–67)	2/10 (inluding7)	3/9/0	55	8/40	NR	<10: 3(25)≥10: 9(75)	<0.5: 10(83)≥0.5: 2(17)	NR	4(33)	NR	12(100)	10(83)	NR
	SRT	28	64(59–67)	12/16	13/15/0	60–64	8/40	NR	<10: 13(46)≥: 15(54)	<0.5: 9(32)≥0.5: 19(68)	NR	4(14)	NR	19(68)	21(75)	NR
Wadasakiet al.[30]	ART	15	66(56–76)	7/5/3	NR	60–66	57/57	1(1–3)	18.7(5.3–71.3)	<0.1	87	6(40)	12(80)	8(53)	6(40)	1(7)
	SRT	42	69(57–76)	20/13/9	NR	60–66	57/57	14(4–78)	12.7(0.9–50.7)	0.7(0.25–2.8)	61	9(21)	20(48)	17(40)	22(52)	0(0)
TAYLORet al.[2]	ART	75	60	9/35/30	27/48/0	60(51–70)	NR	NR	NR	<0.1	88	13(17)	45(60)	73(97)	2(3)	2
	SRT	71	61	18/27/24	12/59/0	70(60–78)	NR	NR	NR	0.6	66	16(23)	31(44)	73(97)	35(49)	35
HAGANet al.[34]	ART	69	70.3	NR	33/36/0	61±1.0	NR	2.9±1.9	10.9±5.6	0.86±0.83	79	36(52)	41(59)	46(67)	0	0
	SRT	88	68.3	NR	60/28/0	64±1.2	NR	40.3±11.2	12.0±4.6	4.5±1.62	45	13(15)	60(73)	42(48)	0	0
Dettiet al.[39]	ART	203	65.1±7.3	44/77/82	22/181/0	66.2±4.1	200(99)	NR	19.2±37.5	0.47±1.73	64	0	0	101(50)	28(14)	30(15)
	SRT	104	67.0±6.0	25/26/53	23/81/0	66.8±4.1	98(94)	NR	22.7±17.3	1.73±3.19	33	0	0	24(23)	18(17)	27(26)
OSTet al.[36]	ART	89	63(51–77)	64/25 (inluding7)	21/63/5	74	NR	3(1–13)	10.0(3.0–47.9)	<0.2	85	23(26)	56(63)	68(76)	NR	48(27)
	SRT	89	64(42–75)	64/25 (inluding7)	21/63/5	76	NR	15(3–132)	10.0(3.5–148)	0.8	65	22(25)	64(72)	59(66)	NR	56(31)
Budihartoet al.[38]	ART	130	64.0(47–75)	76/44/10	34/96/0	60(60–66)	130(100)	NR	10.0(0.8–30.8)	<0.2	90	NR	93(72)	46(35)	NR	NR
	SRT	89	64.0(48–78)	29/45/13	31/58/0	66	89(100)	NR	9.0(1.37–159.4)	0.3(0.04–1.96)	65	NR	31|56	52(58)	NR	NR
Trabulsiet al.[37]	ART	96	63.0(47–75)	22/57/17	0/96/0	64.8(59–70)	NR	NR	9.0(1.7–39)	PSA<0.2 ng/mL	73	23	NR	80(83)	NR	NR
	SRT	96	62.0(42–76)	22/57/17	0/96/0	60(50–70)	NR	NR	8.3(1.1–65.9)	0.7(0.2–2)	50	23	NR	80(83)	NR	NR
BARBARA.et al.[40]	ART	258	65(45–78)	NR	39/206/13	70(63–76)	258(100)	4(1–9)	9.2(2.0–90.0)	NR	79.7	NR	NR	156(60.5)	49(19)	NR
	SRT	173	68(47–81)	NR	74/85/4 8	70(48–78)	171(100)	29(6–146)	9.8(1.7–75)	NR	60.5	NR	NR	58(33.5)	30(18)	NR
PACHOLKEet al.[35]	ART	107	65	28/31/48	4/90/13	57(50–65)	NR	NR	9.5	0	80	NR	87(81)	70(65)	38(38)	NR
	SRT	56	66	13/16/25	9/44/2	60(50–65)	NR	NR	15.2	1.2	39	NR	35(64)	30(55)	16(29)	NR
Sasakiet al.[33]	ART	73	66(36–77)	NR	NR	56(20–70)	NR	1.3(0.53–26.8)	NR	NR	93	4	NR	15	NR	4
	SRT	32	68(58–89)	NR	NR	60(40–70)	NR	20.3(0.82–61)	NR	NR	60	10	NR	29	NR	24
Tsienet al.[32]	ART	38	63(43.8–75.7)	11/16/8	2/36	64(59.4–69.0)	38	2.8(0.9–6.3)	11.6(1.1–99.6)	NR	50	15(39)	35(93)	34(89)	NR	0
	SRT	57	64.2(42.1–78.6)	17/27/8	19/38	65(60.0–75.0)	57	27.7(3.3–116.2)	13.3(0.2–120.0)	1.2(0.2–18.4)	35	9(16)	39(68)	27(47)	NR	0
Doet al.[25]	ART	42	NR	33/9 (inluding7)	NR	64.8	NR	NR	27.6	NR	40	21(50)	21(50)	23(55)	NR	0
	SRT	73	NR	61/12 (inluding7)	NR	64.8	NR	NR	24.9	2.8	26	27(37)	41(56)	36(49)	NR	0
Cattonet al.[24]	ART	54	NR	51/3 (inluding7)	NR	60	NR	NR	NR	NR	81	19(35)	22(41)	52(96)	NR	0
	SRT	59	NR	50/9 (inluding7)	NR	60	NR	NR	NR	2.8	30	17(29)	23(39)	42(72)	NR	0
Valicentiet al.[23]	ART	52	NR	46/7 (inluding7)	NR	64.8	NR	NR	10.0	NR	81	15(29)	37(71)	44(85)	NR	0
	SRT	27	NR	14/13 (inluding7)	NR	64.8	NR	NR	12.0	NR	25	14(52)	13(48)	15(56)	NR	0
Viciniet al.[27]	ART	38	66(51–75)	NR	NR	59.4(50.4–61.2)	NR	NR	8(2–54)	NR	67	NR	NR	24(62)	NR	0
	SRT	23	65(52–79)	NR	NR	61.2(59.4–68)	NR	NR	10(4–60)	NR	16	NR	NR	23(100)	NR	0
Nudellet al.[28]	ART	36	60.3	13/18/5	9/27/0	66.33(55–73)	NR	3.7	9.3	NR	57	11(30)	26(71)	35(97)	NR	0
	SRT	69	NR	54/15 (inluding7)	NR	67.3	NR	NR	14.2	1.0	54	20(29)	48(69)	55(80)	NR	0
Mayeret al.[26]	ART	29	64	NR	NR	70(60–70.4)	NR	2.1(1.4–3.9)	11.9(4.1–166.0)	0.3(0.0–0.4)	85.2	NR	NR	7(24)	NR	6(21)
	SRT	37	66	NR	NR	70	NR	NR	NR	NR	34	NR	NR	4(11)	NR	11(30)

Values are given as mean±s.d./median (range) ART = adjuvant radiotherapy; SRT = salvage radiotherapy; NR = not reported; BFFS: Biochemical Failure-Free Survival; PSM: Positive surgical margins; ECE: extracapsular extension; SVI: seminal vesicle invasion.

### 3. Qualities of included studies

The agreements of the reviewers for selection and validity assessment of the studies were scored by the kappa coefficient (a measure of agreement), which were 0.86 with 93.2% observed agreement and 0.81 with 91.7% observed agreement, respectively. The risks of bias were evaluated by a modification of the Newcastle–Ottawa scale (Table S2 in File S1). Full-length articles were all available for review. Matching criteria between the groups were variable, and little matching information was identified from the conference abstracts. Methods for handling missing data and intention-to-treat analyses were not adequately described in the majority of studies.

### 3. Primary outcomes

#### 3.1 5-year BFFS

**Figure 2:**
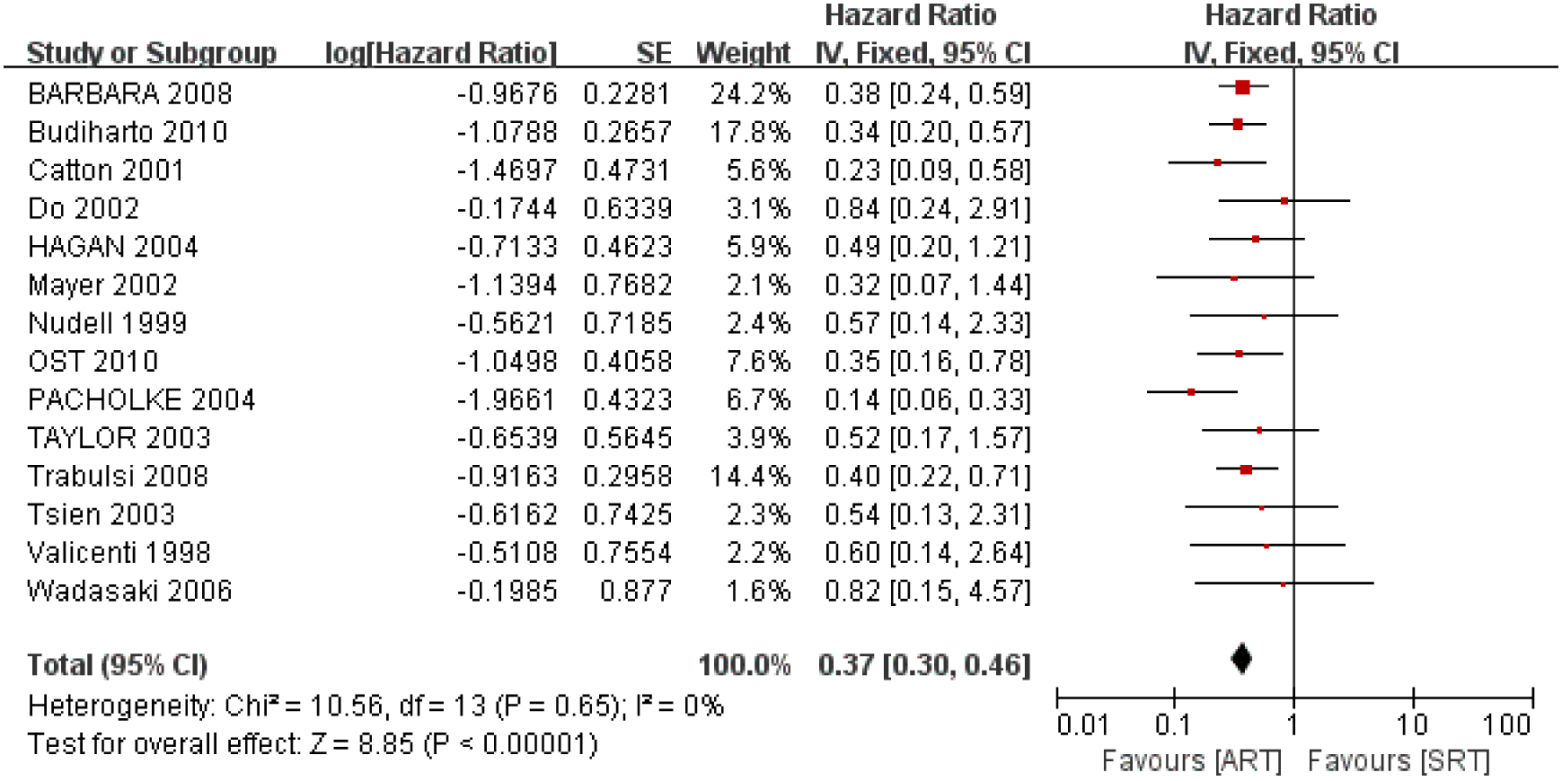
Forest plot for Biochemical Failure-Free Survival (BFFS): 5-years BFFS. ART: adjuvant radiotherapy; SRT: salvage radiotherapy.

**Figure 3:**
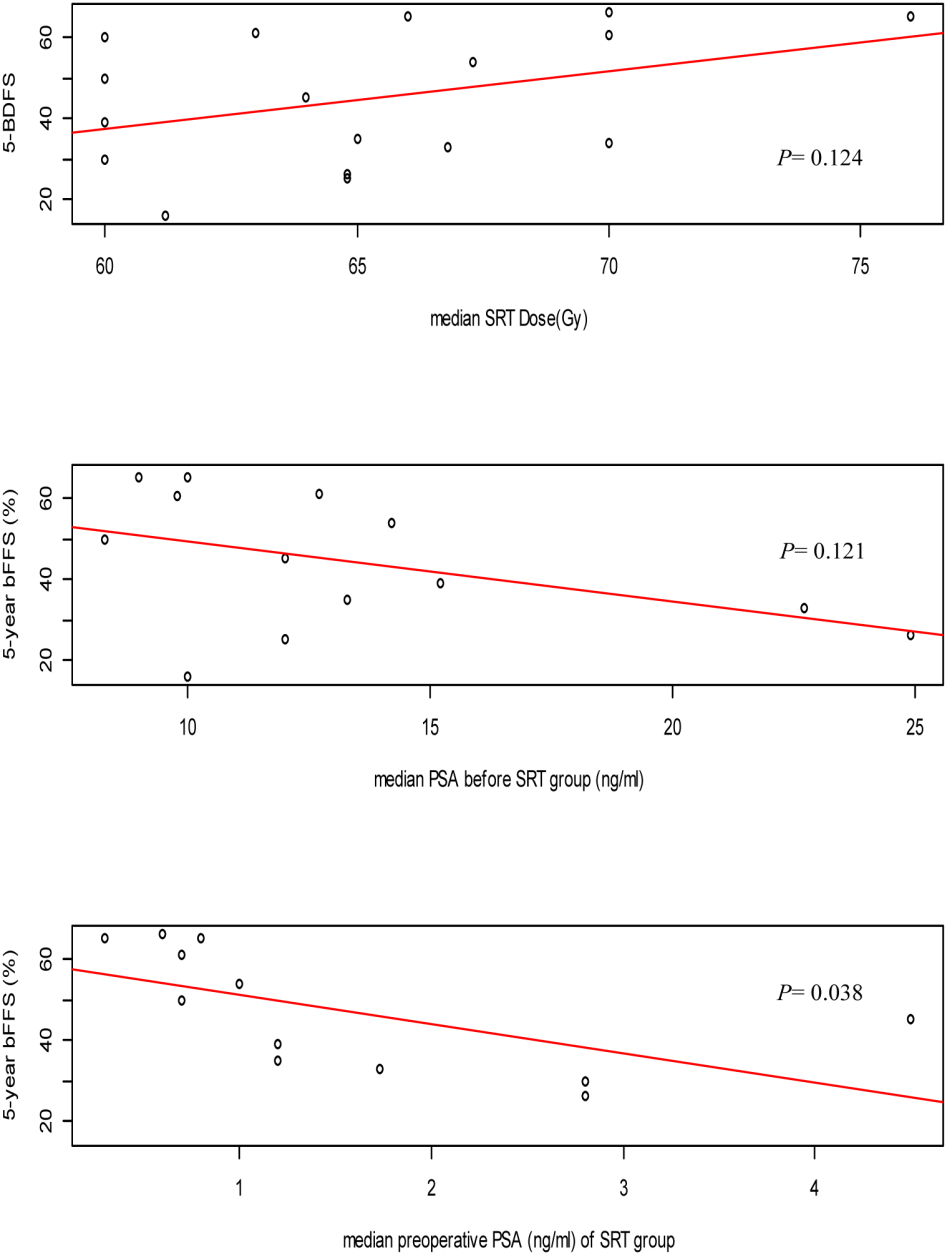
Scatter plots of 5-year biochemical failure-free survival (BFFS) against median salvage radiotherapy (SRT) dose, median PSA before SRT group (ng/ml) and median preoperative PSA of SRT group (ng/ml). (Dotted lines represent results of simple linear regression).

Among the 18 clinical trials included in the meta-analysis, 14 reported HR for 5-year BFFS and the corresponding 95% CIs. These studies assessed 5-year BFFS in 2413 patients showed clearly significant difference between the ART and SRT (HR: 0.37; 95% CI: 0.30–0.46; *p*<0.00001, I^2^ = 0%), which represents a 63% decrease in 5-year BFFS with ART compared to SRT (Figure 2). 5-year BFFS following ART ranged from 52% to 84% (median: 68%) and SRT ranged from 22% to 60% (median: 41%). Scatter plots of 5-year BFFS against median RT dose, PSA at the time of RT and preoperative PSA are shown respectively (Figure 3 and Fig. S1 in File S1). It should be noted that, only median preoperative PSA of SRT group had statistical significance (*p* = 0.038). This simple linear regression was utilized to demonstrate 5-year BFFS decreased with preoperative PSA of SRT group.

#### 3.2 3-year BFFS

**Figure 4:**
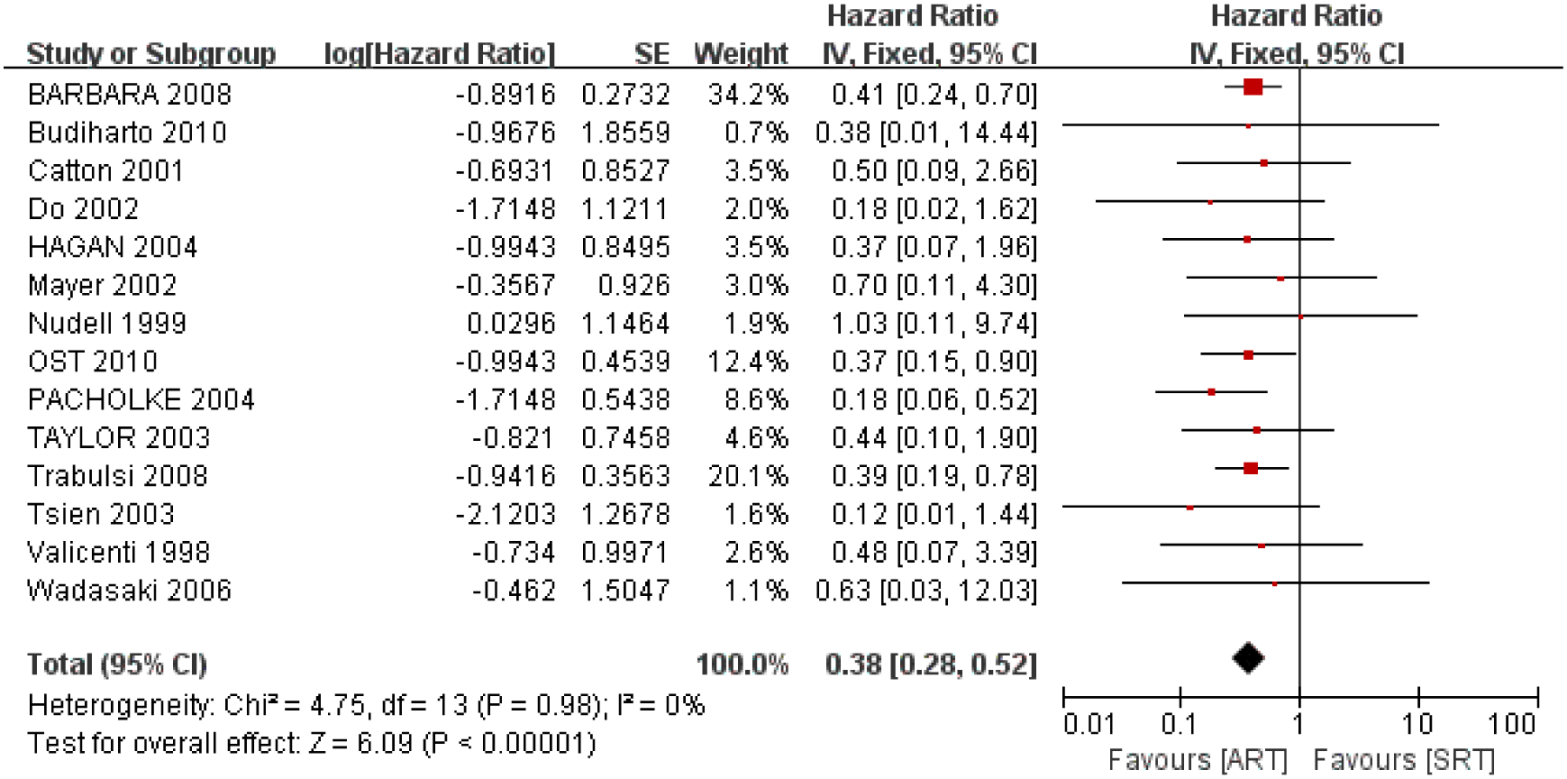
Forest plot for Biochemical Failure-Free Survival (BFFS): 3-years BFFS.

All 14 trials provided data on this endpoint, and the definitions of biochemical failure used by the trials were similar. All 14 trials detected longer biochemical progression-free survival with ART compared with SRT that was statistically significant. Pooling the results of the trials in a meta-analysis (Figure 4) produced an HR of 0.38 (95% CI: 0.28–0.52; *p*<0.00001, I^2^ = 0%), which represents a 62% decrease in 3-year BFFS with ART compared to SRT.

#### 3.2 Overall Survival Rate

**Figure 5:**
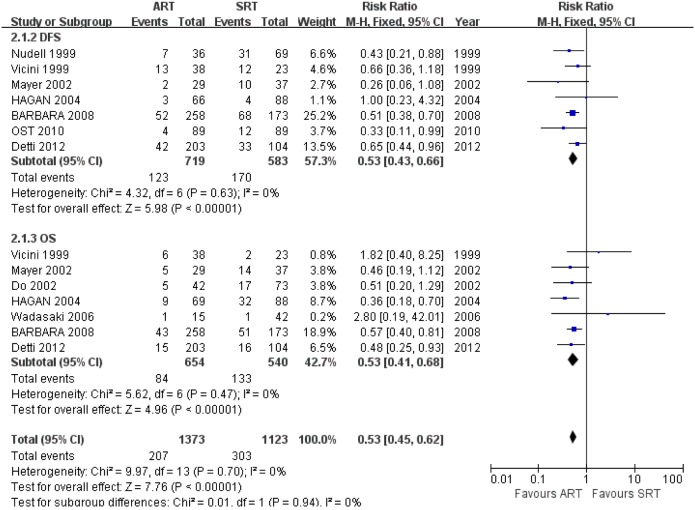
Forest plot for Survival Rate: Disease-free Survival (DFS); overall survival (OS).

All the studies evaluating OS presented a significant difference between the ART and SRT (RR: 0.53; 95% CI, 0.41–0.68; *p*<0.000010) (Fig. 5).Studies evaluating OS presented no evidence of significant heterogeneity between the ART arm and SRT arm (I^2^ = 0%, *p* = 0.47). Sensitivity analysis removing individual studies show clinical heterogeneity of being caused by 2 studies [30], [33] and the result of the other 4 trials demonstrated the advantage of ART (OR: 0.49; 95% CI: 0.38–0.64; *p*<0.001, I^2^ = 0%) (Table.2).

#### 3.3 Disease-Free Survival Rate

Pooling the data of 7 studies consisting of 1302 patients that demonstrated DFS indicated ART was significant better than SRT (RR: 0.53; 95% CI, 0.43–0.66; *p*<0.00001, I^2^ = 0%). We performed a sensitivity analysis including only 6 high-quality studies [29], [30], [31], [37], [39], [42]. The results were similar to the original analysis (RR: 0.52; 95% CI, 0.43–0.65; *p*<0.00001, I^2^ = 0%) (Figure 5).

### 4. Secondary outcomes

#### 4.1 Metastasis-Free Survival Rate

Although not an outcome of interest to this review, we collected data from 5 studies including a total of 472 patients that reported MFS rate showed no significance difference between ART and SRT (RR: 0.81; 95% CI, 0.48–1.36; *p* = 0.43, I^2^ = 0%). Sensitivity analysis by removal of individual studies did not indicate heterogeneity of being caused by a single study (Fig. S2 in File S1).

### 5. Subgroup analysis

#### 5.1 ART versus SRT for 3-year BFFS, 5-year BFFS in age ≥65 years old and age <65 years old

The results showed that biochemical failure free survivals in ART arm had improved 5-year BFFS in patient both younger and older than 65 year old (HR: 0.38; 95% CI, 0.28–0.53; *p*<0.001, I^2^ = 0%; HR: 0.34; 95% CI, 0.24–0.48; *p*<0.001, I^2^ = 0%). The pooling the data of 3-year BFFS and 5-year BFFS both showed no significant difference between two groups (HR: 0.37; 95%C I, 0.27–0.51, *p* = 0.99, I^2^ = 0%; HR: 0.36; 95% CI, 0.29–0.46; *p* = 0.65, I^2^ = 0%)(Fig. S3, S4, S5 in File S1).

#### 5.2 ART versus SRT for OS, 3-year BFFS and 5-year BFFS in Asia, Europe, and Northern America

In subgroup meta-analyses performed separately, there were no significant differences in this subgroup analysis compared with the original analysis. 11 studies reported 3-year BFFS and 7 studies reported OS both showed no significant difference between groups (HR: 0.37; 95% CI, 0.26–0.54; *p* = 0.51, I^2^ = 0%; RR: 0.53; 95% CI, 0.41–0.68; *p* = 0.47, I^2^ = 0%) (Fig. S6, S7, S8 in File S1). Similarly, 5-year BFFS presented no significant difference between groups (HR: 0.37; 95% CI, 0.30–0.46; *p* = 0.89, I^2^ = 0%) (Table 3).

**Table 3:** Subgroup Analysis for Survival Rate.

Group	Pooled analysis (17 studies)				
	Studies	Mean difference(95% CI)	Z	P	I^2^ (%)
Age					
3-yearBFFS(Age <65 y; ≥65 y)	10 Articles (n = 1704)	0.37(0.21–0.57)	5.91	0.95	0
5-year BFFS(Age <65 y; ≥65 y)	10 Articles(n = 1704)	0.36(0.29–0.46)	8.43	0.63	0
DFS (Age <65 y; ≥65 y)	5 Articles (n = 1043)	0.54(0.43–0.67)	5.62	0.16	49.3
District					
3-yearBFFS (District of Asia; Europe; North America)	11 Articles(n = 1621)	0.37(0.26–0.54)	5.25	0.51	0
5-yearBFFS (District of Asia; Europe; North America)	14 Articles(n = 2413)	0.37(0.30–0.46)	8.85	0.65	0
OS (District of Asia; Europe; North America)	7 Articles(n = 1194)	0.53(0.41–0.68)	4.96	0.47	0
Radiation dose					
3-yearBFFS (Radiation dose <70 Gy; ≥70 Gy)	14 Articles(n = 2413)	0.38(0.28–0.52)	6.09	0.59	0
5-yearBFFS (Radiation dose <70 Gy; ≥70 Gy)	14 Articles(n = 2413)	0.37(0.30–0.46)	8.85	0.65	0
OS (Radiation dose <70 Gy; ≥70 Gy)	7 Articles(n = 1194)	0.53(0.41–0.68)	4.96	0.47	0
DFS (Radiation dose <70 Gy; ≥70 Gy)	7 Articles(n = 1302)	0.53(0.43–0.66)	5.98	0.63	0
Publication year					
3-yearBFFS (Publication year 1998–2005; 2006–2010)	11 Articles(n = 1432)	0.38(0.26–0.55)	5.04	0.83	0
5-yearBFFS (Publication year 1998–2005; 2006–2010)	11 Articles(n = 1854)	0.36(0.29-.045)	8.73	0.54	0

BFFS: Biochemical Failure -Free Survival; DFS: disease-free survival; OS: overall survival.

#### 5.3 ART versus SRT for OS, DFS, 3-year BFFS and 5-year BFFS in radiation dose <70 Gy and radiation dose ≥70 Gy

The results indicated that there were no significant differences in this subgroup analysis compared with the original analysis in OS (RR: 0.53; 95% CI, 0.41–0.68; *p* = 0.47, I^2^ = 0%), DFS (RR: 0.53; 95% CI, 0.43–0.66; *p* = 0.63, I^2^ = 0%), 3-year BFFS (HR: 0.38; 95% CI, 0.28–0.52; *p* = 0.59, I^2^ = 0%) and 5-year BFFS (HR: 0.37; 95% CI, 0.30–0.46; *p* = 0.98, I^2^ = 0%) (Fig. S9-S12 in File S1).

#### 5.3 ART versus SRT for 3-year BFFS and 5-year BFFS in publication year from 1998 to 2005 and from 2006 to 2010

The pooling data demonstrated that there were similar results that ART group was significant better than SRT in this subgroup analysis compared with the original analysis in biochemical failure free survival. However, 3-year and 5-year BFFS presented no significant difference between groups (HR: 0.37; 95% CI, 0.30–0.46; *p* = 0.98, I^2^ = 0%; HR: 0.37; 95% CI, 0.30–0.46; *p* = 0.98, I^2^ = 0%) (Fig. S13–S14 in File S1).

### 6. Sensitivity analysis and Publication bias

**Figure 6:**
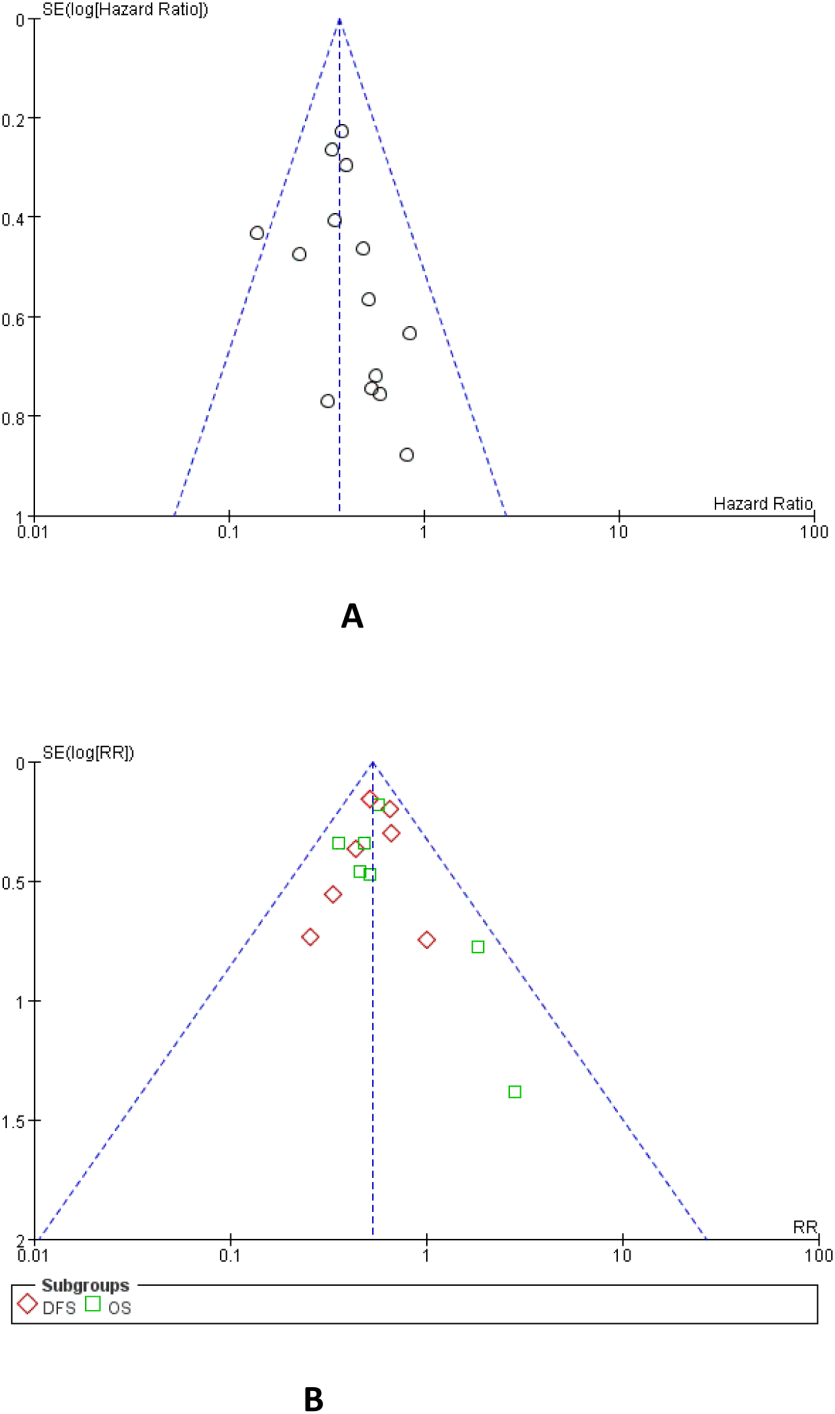
Funnel plots of studies included in the meta-analysis: A) 5-year BFFS; B) Survival Rate (DFS; OS).

2 matched control studies and 16 retrospective studies that scored five or more stars on the modified Newcastle-Ottawa scale were included in sensitivity analysis (Table 4). There was no change in the significance of any of the outcomes except for 3-year and 5-year BFFS, which was shown that the heterogeneity obviously decreased. We applied funnel plots to evaluate publication bias of the included studies. All of the funnel plots were symmetrical. All studies lie inside the 95% CIs, with an even distribution around the vertical, indicating no obvious publication bias (Figure 6).

**Table 4:** Results of meta-analysis comparison of ART and SRT.

Outcomesof interest	Overall analysis									Sensitivity analysis								
	Studies, no.	ARTpatients, no.	SRTpatients, no.	HR¶/RR(95% CI)	*p* value	Studyheterogeneity				Studies, no.	ARTpatients, no.	SRTpatients, no.	HR/RR(95% CI)	*p* value	Studyheterogeneity			
						X^2^	df	I^2^,%	*p* value						X^2^	df	I^2^,%	*p* value
***Primary outcomes***																		
3-year BFFS¶	14	1090	1026	0.38(0.28–0.52)	<0.001	4.75	13	0	0.98	11	948	871	0.36(0.26–0.50)	<0.001	3.76	10	0	0.96
5-year BFFS¶	14	1090	1026	0.37(0.30–0.46)	<0.001	10.56	13	0	0.65	10	906	798	0.37(0.29–0.47)	<0.001	7.43	9	0	0.56
OS	7	654	540	0.53(0.41–0.68)	<0.001	5.62	6	0	0.47	5	601	475	0.49(0.38–0.64)	<0.001	1.47	4	0	0.83
DFS	7	719	583	0.53(0.43–0.66)	<0.001	4.32	6	0	0.63	6	653	495	0.52(0.43–0.65)	<0.001	3.67	5	0	0.60
***Secondary outcomes***																		
MFS	5	209	263	0.81(0.48–1.36)	0.43	2.51	4	0	0.64	4	180	226	0.76(0.45–1.29)	0.31	0.62	3	0	0.89

ART = adjuvant radiotherapy; SRT = salvage radiotherapy; BFFS: Biochemical Failure-Free Survival; DFS: disease-free survival; OS: overall survival; MFS: Metastasis-free survival HR: hazard ratio; RR: risk ratio; df = degrees of freedom; CI = confidence interval; ¶: Included studies applied HR; : Included studies applied RR.

## Discussion

In 2014, an estimated 233000 men were diagnosed with prostate cancer and prostate cancer surpassed lung cancer as the most common cancer in man [43]. In approximately two-thirds of men, radical prostatectomy constitutes a cure but within 10 years up to one-third of patients manifest recurrent disease [44], [45]. When the patients with localized prostate cancer who undergo a radical prostatectomy (RP) will remain disease free, patients with APFs were known to be at an increased risk for developing a biochemical recurrence and distant metastatic disease. APFs that have been significantly associated with an increased chance for a biochemical recurrence include higher preoperative prostate-specific antigen (PSA), extracapsular extension (ECE), seminal vesicle invasion (SVI) and positive surgical margins (PSM). There were currently two treatment approaches for the radiotherapy of patients with APFs following a RP, largely based upon differences in physician beliefs and practices of ART between treating to before radiation oncologists and urologists [15]. One approach is to treat all patients with APFs with ART, based upon information from pathological review of the RP specimen in men with an undetectable PSA (typically <0.2 ng/ml). The other approach is to closely follow patients with serial PSA readings and initiate the SRT once a PSA failure is detected. Three randomized trials have examined the rate of BFFS in patients treated with ART versus observation so far [3], [46], [47]. Nevertheless, we focused on the real clinically relevant question which was whether RT should be administered immediately after prostatectomy or postponed until first occurrence of PSA rise. Previous literature couldn’t answer this question because existing studies assessing the value of SRT were conducted in patients with advanced PSA recurrence (median PSA: 0.5 ng/ml)[47], [48], [49]. Additional, only half actually received SRT for those treatment failures after radical prostatectomy [46]. Given the absence of data comparing ART and SRT, we sought to examine this issue.

This meta-analysis of 2 matched control studies and 16 retrospective studies including a total of 2629 cases were identified (1404 cases for ART and 1185 cases for SRT) comparing the efficacy of ART and SRT, which have demonstrated 3-year and 5-year BFFS, DFS advantages in favor of ART. The most valuable finding of our study has revealed an overall survival benefit of ART. We also found that MFS was considerable and no significant differences in both two groups. Hence, we reported the analysis of the impact of ART and SRT on survival after RP. It was well established that early ART provides improved OS, BFFS, DFS for patients with APFs following a prostatectomy compared with SRT. Moreover, initial observation followed by SRT in cases of relapse is equivalent to ART remains unknown.

Overall survival is certainly the outcome of greatest importance for any cancer therapy as it incorporates them effect of mortality secondary to cancer, the interventions used, and all other causes. Given the relatively natural history of prostate cancer, it is anticipated that lengthy follow-up is necessary to assess differences in OS. With regard to the RCTs of ART, SWOG 8794 and EORTC 22911, OS was less clear, with benefits reported in SWOG 8794 with long-term data [31]. Our study included 7 clinical trials that demonstrated the OS benefit with ART and ART following RP in patients with APFs prolong the OS compared to SRT. The other primary outcome of the study was BFFS. Biochemical recurrence as a detectable or rising PSA value after surgery that is >0.2 ng/ml with a second confirmatory level >0.2 ng/ml. With a median follow-up of 3 and 5 years, a significant improvement in BFFS was noted for the ART arm. In summary, all the included studies of ART versus SRT demonstrated improved in outcomes in patients after RP with APFs who received ART.

To better determine possible relationships between ART and SRT, the subgroup analyses were conducted according to age, district, and radiation dose and publication year. Our exploratory subgroup analyses revealed some interesting, hypothesis-generating findings. The 3-year, 5-year BFFS and DFS for ≥65 years age group were similar with <65 years age group. In the district arm, there were no significance differences in 3-year BFFS, 5-year BFFS and OS. Furthermore, we observed the radiation dose with <70 Gy comparing ≥70 Gy between ART and SRT, suggesting that the ≥70 Gy group was equivalent to <70 Gy group in 3-year and 5-year BFFS, OS, DFS. Based on our analysis, we don’t recommend delivering a higher equivalent dose for patients after RP through a shorter course and larger fraction RT schedules. Finally, we hypothesized that the efficiency of ART and SRT reported in men after RP may have increased over the past decade because of the improvement of imaging and RT techniques. Therefore, we explored the impact of publication year on 2 matched control studies and 16 retrospective studies. However, the 5-year BFFS arm and 3-year BFFS arm demonstrated that the publication year didn’t reach statistical significance.

Assuming that the residual tumor burden after prostatectomy would be many logarithms smaller than in the case of radical irradiation, the dose of radiotherapy used in the postoperative setting was generally 20–25% lower (around 60 vs. 76–80 Gy) than that commonly used in the case of radical irradiation [50], but both the appropriate dose for ART and SRT, and whether patients benefited from increasing the dose remain unclear so far. Of the included studies, the dosage of radiotherapy also varied widely. Therefore, we introduced simple linear regression to analyze the relationship between RT dose and prognosis of the patients, revealing that the 5-year BFFS was improved by increasing RT dose in patients receiving SRT. However, in patients receiving ART, the 5-year BFFS was not statistical change with the RT dose. Our results were in consists with the previous studies which had described the higher SRT dose might improve the 5-year BFFS in single-institution [51], [52] and multi-institutional [53], [54]. Besides the relationship between RT dose and survival benefits, we also analyzed the predictive values of pre-operative and pre-RT PSA on 5-year BFFS. Our results demonstrated that patients with high PSA burden, no matter preoperative or pre-RT all indicated poor prognosis. This finding was in accordance with the results of most previous studies [55], [56]. It was worth noting that increased PSA value of patients receiving SRT revealed a more obvious correlation with decreased 5-year BFFS compared that of patients receiving ART. Previous analyses by King et al had separately described the importance of SRT dose and pre-SRT PSA [24], [57]. It was possible that high PSA is an indicator of aggressive disease that was less likely to be cured by SRT.

Currently two cooperative groups with Phase III trials will hopefully help to determine the appropriate timing for RT in the postoperative setting. The RADICALS trial administered by the Medical Research Council in England, is randomizing patients with APFs to ART or SRT (initiated following two consecutive PSA rises >0.1 ng/ml or three consecutive PSA rises. The second study, a Phase III trial conducted by Trans-Tasman Radiation Oncology Group randomize patients to ART, initiated within 4 months following a RP or early SRT initiated once the PSA levels are ≥0.2 ng/ml. The primary end point of this study will be PSA failure. However, data from these trials are not expected to become available for another decade. In the meantime, there is no consensus on whether patients should be treated with ART or SRT. The study of this system review and meta-analysis focus on this issue, hopefully providing the important advice to answer this question.

The present meta-analysis has the following limitations that must be taken into account. The main limitation is that all the included studies were retrospective samples. Inadequate random sequence generation and blinding tended to increase the risk of bias. Secondly, the other main limitation is lack of toxicity comparison between ART and SRT. After administrating post-RT, possible short-term and long-term urinary, bowel and sexual side effects might occur. However, due to long time span and lack of necessary information on the SRT toxicity within the multi-institutional database did not allow us to compare differences in such an endpoint between the two treatment approaches. Moreover, few study focused on the use of androgen deprivation therapy (ADT) in patients who underwent prostatectomy and then ART or SRT. It is difficult to accumulate data regarding the use of ADT in conjunction with RT. As it is the key point, randomized controlled trials are needed to provide definitive evidence. Finally, the included literature doesn’t reflect implementation of these newer methods, with only one-quarter of the studies (6/18) reporting use of 3D-CRT techniques and even less reporting use of IMRT techniques.

Nevertheless, this meta-analysis was conducted at an appropriate time, because enough data have accumulated for inspection by meta-analytical methods and we reach the conclusions that reported 3-year and 5-year BFFS, OS and DFS indicated that ART might reduce the need for SRT. We applied multiple strategies to identify studies, strict criteria to include and evaluate the methodological quality of the studies, and subgroup and sensitivity analysis to minimize the heterogeneity. Thus, we provided the most update information in this area.

## Conclusions

This meta-analysis indicates that ART therapy offers a safe and efficient alternative to SRT with longer 3-year and 5-year BFFS, better OS and DFS. Our recommendation is to suggest ART for all patients with APFs and may reduce the need for SRT. Given the inherent limitations of the included studies, future well-designed RCTs are awaited to confirm and update the findings of this analysis.

## Supporting Information

Checklist S1 **PRISMA Checklist.**
(DOC)

File S1 **Supplementary data: Table S1**. Study Eligibility Criteria for Inclusion in the Review. **Table S2**. Risk of bias in retrospective studies using modified (Newcastle-Ottawa scale). **Figure S1** Scatter plots of 5-year biochemical failure-free survival (BFFS) against median salvage radiotherapy (ART) dose, median PSA before ART group (ng/ml) and median preoperative PSA of ART group (ng/ml). (Dotted lines represent results of simple linear regression). **Figure S2** Forest plot for Metastasis-free survival (MFS). **Figure S3** Forest plot for 3-year BFFS in subgroup analysis A) age of included patients was <65 years old B) age ≥65 years old. **Figure S4** Forest plot for 5-year BFFS in subgroup analysis A) age of included patients was <65 years old B) age ≥ than 65 years old. **Figure S5** Forest plot for Disease Free Survival in subgroup analysis A) age of included patients was younger than 65 years old B) age wasn’t younger than 65 years old. **Figure S6** Forest plot for 3-year BFFS in subgroup analysis A) district of included patients was Northern American; B) district of patient was Asian; C) district of patient was European. **Figure S7** Forest plot for 5-year BFFS in subgroup analysis A) district of included patients was European; B) district of patient was Asian; C) district of patient was Northern American. **Figure S8** Forest plot for Overall Survival in subgroup analysis A) district of included patients was Asian; B) district of patient was European; C) district of patient was Northern American. **Figure S9** Forest plot for 3-year BFFS in subgroup analysis A) radiation dose of included patients was <70 Gy; B) radiation dose of included patients was ≥70 Gy. **Figure S10** Forest plot for 5-year BFFS in subgroup analysis A) radiation dose of included patients was <70 Gy; B) radiation dose of included patients was ≥70 Gy. **Figure S11** Forest plot for Disease Free Survival in subgroup analysis A) radiation dose of included patients was <70 Gy; B) radiation dose of included patients was ≥70 Gy **Figure S12** Forest plot for Overall Survival in subgroup analysis A) radiation dose of included patients was <70 Gy; B) radiation dose of included patients was ≥70 Gy. **Figure S13** Forest plot for 3-year BFFS in subgroup analysis A) publication year (1998–2005); B) publication year (1998–2005). **Figure S14** Forest plot for 5-year BFFS in subgroup analysis A) publication year (1998–2005); B) publication year (1998–2005).
(DOC)
