# Supplementary material for: Adjuvant and Salvage Radiotherapy after Prostatectomy: A Systematic Review and Meta-Analysis
Source: PLoS One. 2014 Aug 14;9(8):e104918. doi: 10.1371/journal.pone.0104918 (PMC4133270; doi:10.1371/journal.pone.0104918)
Supplement: File S1 — Supplementary data: Table S1. Study Eligibility Criteria for Inclusion in the Review. Table S2. Risk of bias in retrospective studies using modified (Newcastle-Ottawa scale). Figure S1 Scatter plots of 5-year biochemical failure-free survival (BFFS) against median salvage radiotherapy (ART) dose, median PSA before ART group (ng/ml) and median preoperative PSA of ART group (ng/ml). (Dotted lines represent results of simple linear regression). Figure S2 Forest plot for Metastasis-free survival (MFS). Figure S3 Forest plot for 3-year BFFS in subgroup analysis A) age of included patients was <65 years old B) age ≥65 years old. Figure S4 Forest plot for 5-year BFFS in subgroup analysis A) age of included patients was <65 years old B) age ≥ than 65 years old. Figure S5 Forest plot for Disease Free Survival in subgroup analysis A) age of included patients was younger than 65 years old B) age wasn’t younger than 65 years old. Figure S6 Forest plot for 3-year BFFS in subgroup analysis A) district of included patients was Northern American; B) district of patient was Asian; C) district of patient was European. Figure S7 Forest plot for 5-year BFFS in subgroup analysis A) district of included patients was European; B) district of patient was Asian; C) district of patient was Northern American. Figure S8 Forest plot for Overall Survival in subgroup analysis A) district of included patients was Asian; B) district of patient was European; C) district of patient was Northern American. Figure S9 Forest plot for 3-year BFFS in subgroup analysis A) radiation dose of included patients was <70 Gy; B) radiation dose of included patients was ≥70 Gy. Figure S10 Forest plot for 5-year BFFS in subgroup analysis A) radiation dose of included patients was <70 Gy; B) radiation dose of included patients was ≥70 Gy. Figure S11 Forest plot for Disease Free Survival in subgroup analysis A) radiation dose of included patients was <70 Gy; B) radiation dose of included patients was ≥70 Gy Figure S12 [file pone.0104918.s002.doc]

**Supplementary**

| **Table S1. Study Eligibility Criteria for Inclusion in the Review*** | |
| --- | --- |
| **Participates** | **Adult Patients Requiring Radical Prostatectomy** |
| **Types of interventions**  **Types of outcome**  **measures** | **All studies evaluating adjuvant radiotherapy(ART) after** **radical prostatectomy, deﬁned as**  1.Patients must have at least one of the following risk factors: 1) Positive margins, 2) Extra prostatic extension (EPE) with or without seminal vesicle involvement (pT3a or pT3b),3) lymph node invasion(LNI)  2. Patients were irradiated within 6 months of their radical prostatectomy for adenocarcinoma of the prostate.  3. Patients had an undetectable serum PSA at the start of radiation therapy.  4. None received any neo-adjuvant therapy.  **Compared with salvage radiotherapy(SRT) after radical prostatectomy, deﬁned as:**  1.Patients must have at least one of the following risk factors: 1) Positive margins, 2) Extra prostatic extension (EPE) with or without seminal vesicle involvement (pT3a or pT3b) 3) lymph node invasion(LNI)  2. Patients were referred for radiation therapy because of a persistent postoperative serum PSA.  3. Patients manifested a PSA recurrence after a period of undetectable PSA.  4、None received neo-adjuvant therapy.  **All studies including one or more of the following**  **1.Primary outcomes:**  1.5-years BFFS（biochemical failure-free survival）  2.3-years BFFS（biochemical failure -free survival）  3.OS（Overall survival）  4.DFS（disease-free survival）  **2 Secondary Outcome Measures:**  MFS（Metastasis-free survival） |
| *A study must meet the two components above for inclusion in this review. | |

| **Table S2. – Risk of bias in retrospective studies using modified（Newcastle-Ottawa scale）** | | | | | | | | |
| --- | --- | --- | --- | --- | --- | --- | --- | --- |
| Study | Selection | | | Comparability | | Outcome | | Quality  score |
|  | Assignment for  treatment† | Representative  treatment group | Representative  reference group | Comparable for 1,2,3,4,5,6 * | Comparable for 7,8,9,10,11,12,13* | Assessment of  outcome | Adequate  follow-up |
| Hudson et al.[29] | No | Yes | Yes | 1,2,3,4,5,6 | 7,9,11,12, | Yes | No | ★★★★★★ |
| Wadasaki et al.[30] | No | Yes | Yes | 1,2,4,5,6 | 7,8,9,10,11,12,13 | Yes | No | ★★★★★★ |
| TAYLOR et al.[2] | No | Yes | Yes | 1,2,3,4,6 | 9,10,11,12,13 | Yes | Yes | ★★★★★★ |
| HAGAN et al.[34] | No | Yes | Yes | 1,2,3,4,5,6 | 8,9,10,11,12,13 | Yes | Yes | ★★★★★★ |
| Detti et al.[39] | No | Yes | Yes | 1,2,3,4,5,6 | 7,9,10,11,12,13 | Yes | Yes | ★★★★★★ |
| OST et al.[36] | No | Yes | Yes | 1,2,3,4,5 | 8,9,10,11,13 | Yes | Yes | ★★★★★★ |
| Budiharto et al. [38] | No | Yes | Yes | 1,2,3,4,5 | 7,10,11 | Yes | Yes | ★★★★★ |
| Trabulsi et al.[37] | No | Yes | Yes | 1,2,3,4,5,6 | 9,11 | Yes | Yes | ★★★★★ |
| BARBARA. et al. [40] | No | Yes | Yes | 1,3,4,5 | 7,8,11,12 | Yes | No | ★★★★★★ |
| PACHOLKE et al. [35] | No | Yes | Yes | 1,2,4,5,6 | 9,11,12, | Yes | Yes | ★★★★★ |
| Sasaki et al.[33] | No | Yes | Yes | 1,4 | 8,9,11,13 | Yes | No | ★★★★ |
| Tsien et al. [32] | No | Yes | Yes | 1,2,3,4,5 | 7,8,9,10,11,13 | Yes | Yes | ★★★★★★ |
| Do et al.[25] | No | Yes | Yes | 2,4,5,6 | 9,10,11,13 | Yes | Yes | ★★★★★★ |
| Catton et al.[24] | No | Yes | Yes | 2,4 | 9,10,11,13 | Yes | Yes | ★★★★★ |
| Valicenti et al. [23] | No | Yes | Yes | 2,4,5 | 9,10,11,13 | Yes | Yes | ★★★★★ |
| Vicini et al. [27] | No | Yes | Yes | 1,4,5 | 13 | Yes | Yes | ★★★★ |
| Nudell et al.[28] | No | Yes | Yes | 2,4,5 | 9,10,11,13 | Yes | Yes | ★★★★★ |
| Mayer et al. [26] | No | Yes | Yes | 1,4, | 11 | Yes | Yes | ★★★★ |
| Comparability variables: 1 = age; 2 = Gleason score; 3 = Postoperative stage; 4 = Radiation dose; 5 = preoperative PSA; 6 = Pre-RT PSA  7 =3D-CRT; 8 = Interval (months); 9 = Seminal vesicle involvement；10 = Extra capsular extension; 11 = Positive surgical margins; 12 = Preoperative hormones; 13 = Postoperative hormones;  † Details of criteria for adequate random assignment of patients to treatments were provided.  * If all characteristics were comparable, two stars; if four or five characteristics were comparable, one star; otherwise, no star. | | | | | | | | |

*P*= 0.502

*P*= 0.079

*P*= 0.298

**Figure.S1** **Scatter plots of 5-year biochemical failure-free survival (BFFS) against median salvage radiotherapy (ART) dose, median PSA before ART group (ng/ml) and median preoperative PSA of ART group (ng/ml). (Dotted lines represent results of simple linear regression).**


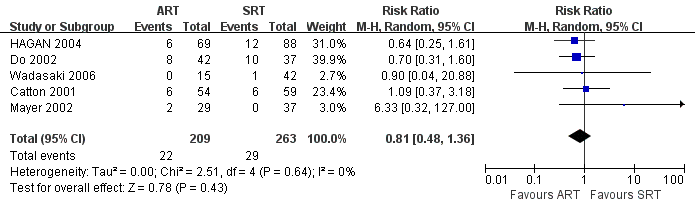


**Figure.S2 Forest plot for** **Metastasis-free survival (MFS)**


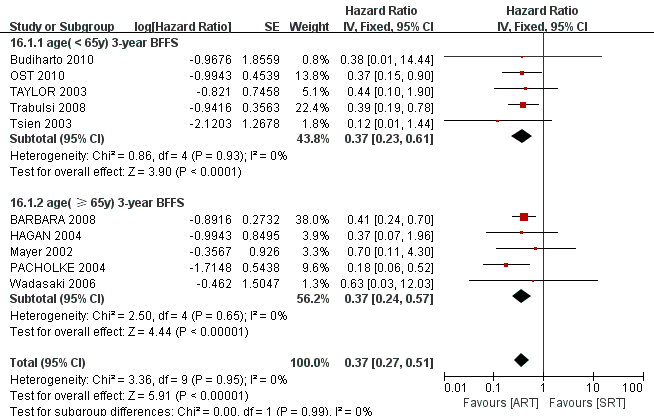


**Figure.S3 Forest plot for 3-year BFFS in subgroup analysis A) age of included patients was < 65 years old B) age ≥ 65 years old**

**
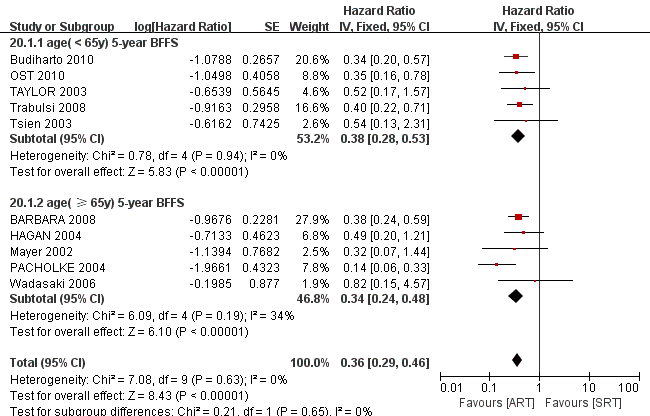
**

**Figure.S4 Forest plot for 5-year BFFS in subgroup analysis A) age of included patients was < 65 years old B) age ≥ than 65 years old**

**
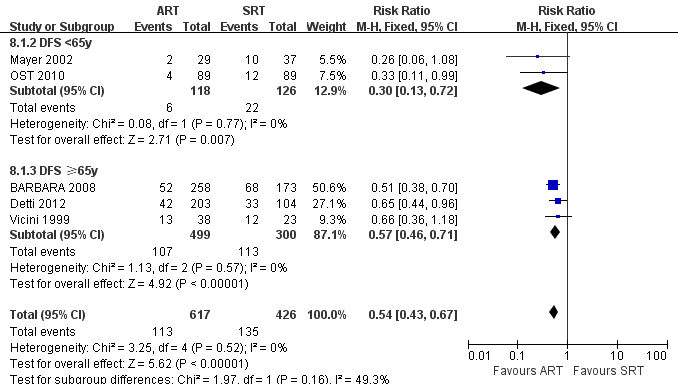
**

**Figure.S5 Forest plot for Disease Free Survival in subgroup analysis A) age of included patients was younger than 65 years old B) age wasn’t younger than 65 years old**

**
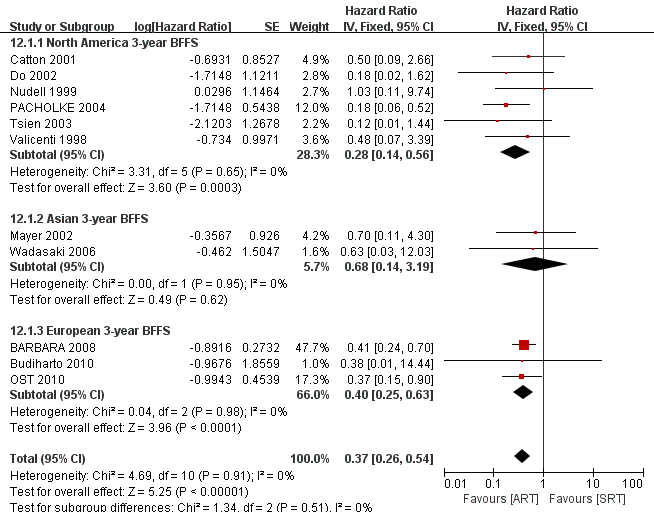
**

**Figure.S6 Forest plot for 3-year BFFS in subgroup analysis A) district of included patients was Northern American; B) district of patient was Asian; C) district of patient was European**

**
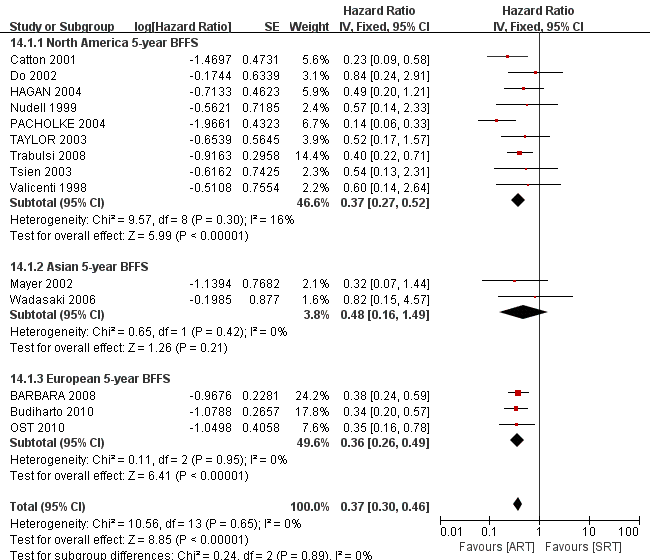
**

**Figure.S7 Forest plot for 5-year BFFS in subgroup analysis A) district of included patients was European; B) district of patient was Asian; C) district of patient was Northern American**

**
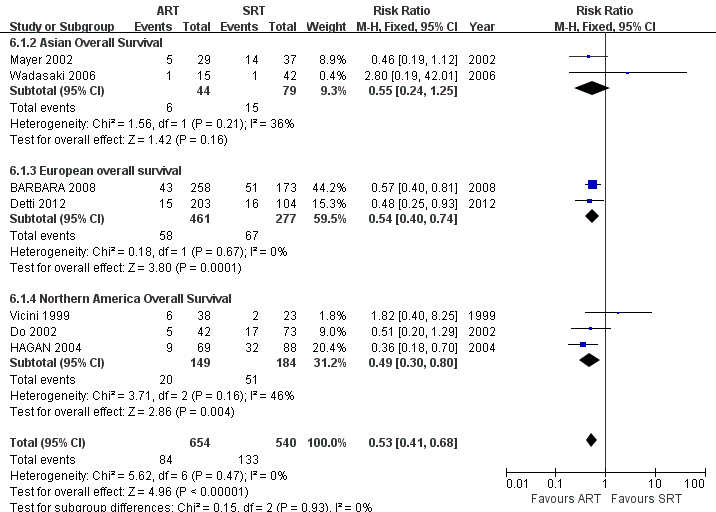
**

**Figure.S8 Forest plot for Overall Survival in subgroup analysis A) district of included patients was Asian; B) district of patient was European; C) district of patient was Northern American**

**
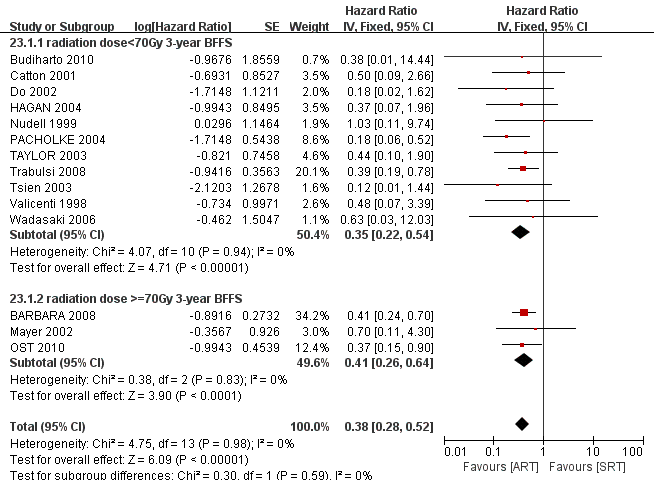
**

**Figure.S9 Forest plot for 3-year BFFS in subgroup analysis A) radiation dose of included patients was <70 Gy; B) radiation dose of included patients was ≥70 Gy**

**
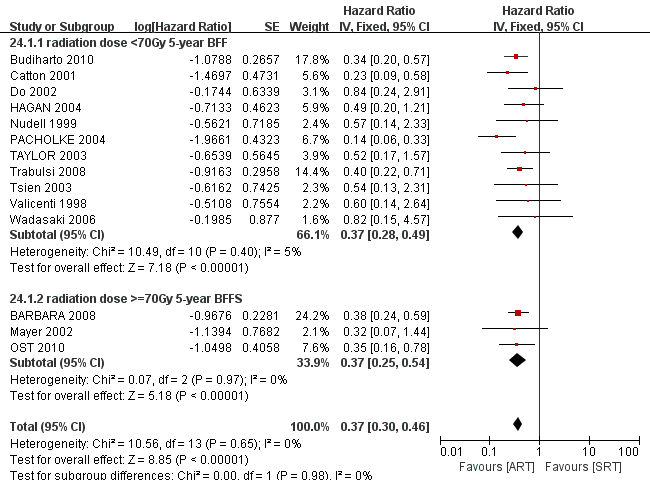
**

**Figure.S10 Forest plot for 5-year BFFS in subgroup analysis A) radiation dose of included patients was <70 Gy; B) radiation dose of included patients was ≥70 Gy**

**
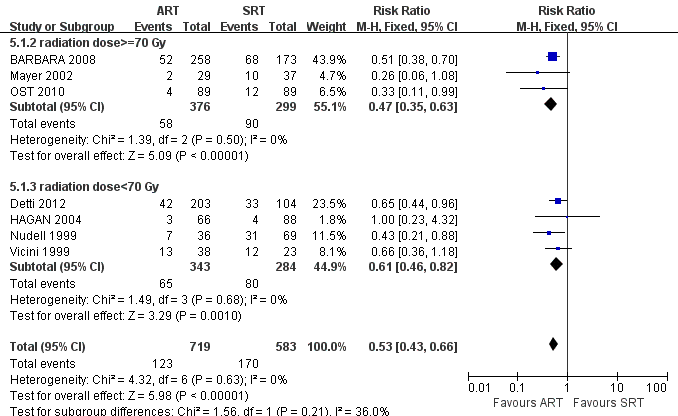
**

**Figure.S11 Forest plot for Disease Free Survival in subgroup analysis A) radiation dose of included patients was <70 Gy; B) radiation dose of included patients was ≥70 Gy**

**
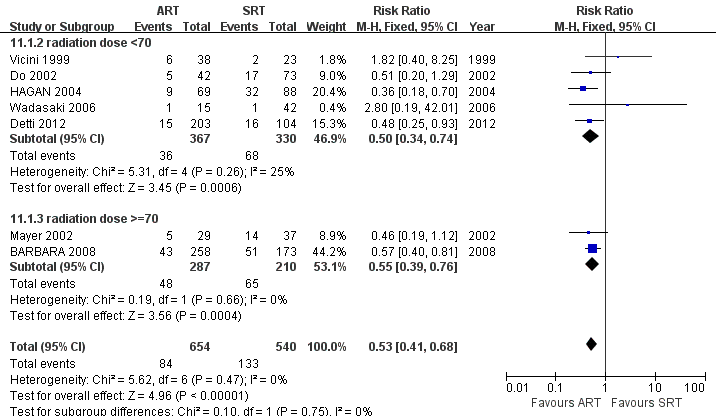
**

**Figure.S12 Forest plot for Overall Survival in subgroup analysis A) radiation dose of included patients was <70 Gy; B) radiation dose of included patients was ≥70 Gy**

**
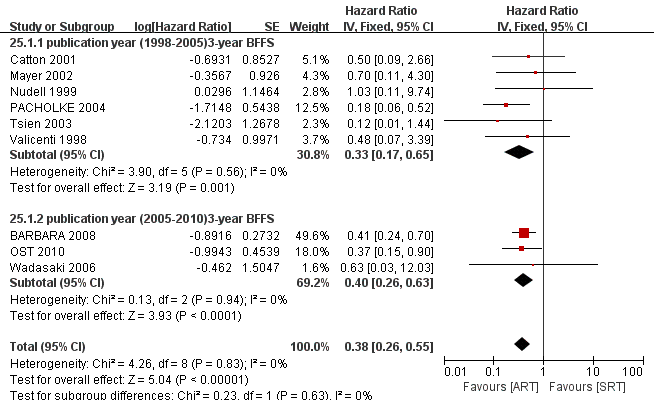
**

**Figure.S13 Forest plot for 3-year BFFS in subgroup analysis A) publication year (1998-2005); B) publication year (1998-2005)**

**
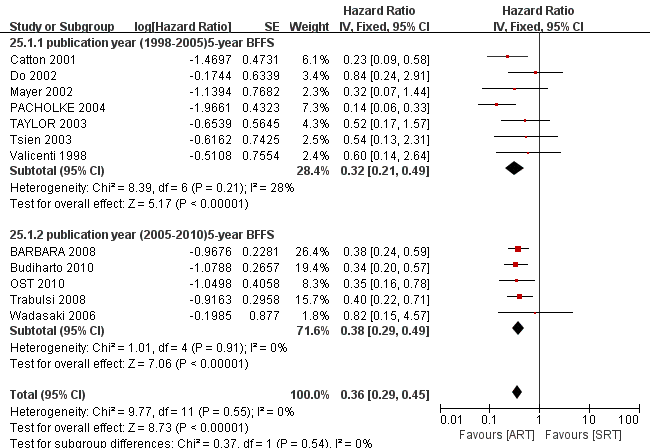
**

**Figure.S14 Forest plot for 5-year BFFS in subgroup analysis A) publication year (1998-2005); B) publication year (1998-2005)**
